# S188. DYSREGULATION OF CIRCULAR RNA EXPRESSION IN SCHIZOPHRENIA OBSERVED IN POSTMORTEM DORSOLATERAL PREFRONTAL CORTEX

**DOI:** 10.1093/schbul/sby018.975

**Published:** 2018-04-01

**Authors:** Ebrahim Mahmoudi, Chantel Fitzsimmons, Michael Geaghan, Murray Cairns

**Affiliations:** School of Biomedical Sciences & Pharmacy, University of Newcastle

## Abstract

**Background:**

The last few years has witnessed the emergence of a novel class of long non-coding RNA known as circular RNA (circRNA). These molecules are characterised by their circularity formed through the back splicing of 3’ and 5’ ends of transcript segments produced by one or more of its exons. CircRNAs function as transcriptional modulators, microRNA regulators, as well as template for translation. In our current study, we profiled circRNA expression in post-mortem brain samples from Schizophrenia (SZ) and control subjects using next-generation sequencing technology to discover the association of these novel RNA molecules with the pathogenesis of SZ.

**Methods:**

Total RNA from cerebral cortex (BA46) of 17 SZ patients and 18 healthy controls were subjected to ribosomal RNA depletion and then RNase R treatment to further deplete linear RNA and enrich for exonuclease resistant circRNA transcripts. Sequencing libraries were constructed using Illumina TruSeq RNA Library Prep Kit (LT) (150 cycles) and sequenced by an Illumina NexSeq500. Sequencing data was analysed by the CIRCexplorer2 pipeline to identify circRNA transcripts. To validate the sequencing findings, real-time PCR was performed using outward primers sets designed to specifically amplify circular transcripts.

**Results:**

We discovered a large number of distinct circRNAs (95,212), many of which were highly expressed throughout the cohort. Surprisingly, a large proportion (52%) of the identified circRNAs sequences were novel or not previously reported. Differential expression analysis suggested that there was substantial alteration in circRNA expression in SZ. More than two thirds of these molecules displayed decreased expression, whereas the remainder were upregulated. Functional annotation of the host genes was significantly enrichment for terms-related to neurobiology and neurocognitive impairment including clusters such as neurogenesis, differentiation and synapse. Many of these circRNAs were also predicted to interact with miRNAs, supporting a potential miRNA sponging function for these circRNA.

**Discussion:**

RNA sequencing in the human postmortem DLPFC revealed dysregulation of circRNA expression in schizophrenia. This alteration was characterized by a substantial decrease in circRNA expression in the disorder. Bioinformatic predictions of circRNA interaction suggest they function as miRNA regulators and may have a broader role in etiology or pathophysiology of the disorder.

